# Supplementary material for: Estimating long-term vaccine effectiveness against SARS-CoV-2 variants: a model-based approach
Source: Nat Commun. 2023 Jul 19;14:4325. doi: 10.1038/s41467-023-39736-3 (PMC10356855; doi:10.1038/s41467-023-39736-3)
Supplement: Supplementary file 1 — Supplementary Information [file 41467_2023_39736_MOESM1_ESM.pdf]

Estimating long-term vaccine effectiveness against SARS-CoV-2 variants: a model-based approach.

Alexandra B Hogan[1,2], Patrick Doohan[2], Sean L Wu[3], Daniela Olivera Mesa[2], Jaspreet Toor[2], Oliver J Watson[2,4], Peter Winskill[2], Giovanni Charles[2], Gregory Barnsley[2,4], Eleanor M Riley[5], David S Khoury[6], Neil M Ferguson[2], Azra C Ghani[2]

1. School of Population Health, Faculty of Medicine and Health, University of New South Wales, Sydney, NSW, Australia
2. MRC Centre for Global Infectious Disease Analysis, School of Public Health, Imperial College London, London, UK
3. Institute for Health Metrics and Evaluation, University of Washington, Seattle, USA
4. London School of Hygiene and Tropical Medicine, London, UK
5. Institute of Immunology and Infection Research, School of Biological Sciences, University of Edinburgh, UK
6. Kirby Institute, University of New South Wales, Sydney, NSW, Australia

Supplementary Data

Table S1: Estimated vaccine effectiveness against mild disease, hospitalisation and death for Oxford/AstraZeneca AZD1222, Pfizer-BioNTech BNT162b2 and Moderna mRNA-1273 vaccine regimens as a function of time since dose 2 or dose 3. Estimates are shown for the Delta variant; estimates for the Omicron variant are shown in **Table 2**. The values are shown for each vaccine for dose 2 or dose 3; as the dose 3 estimates do not depend on dose 2 values, the estimates are applicable to either homologous or heterologous dosing. Values shown are the posterior median and 95% credible intervals. The comparator group is those that did not receive any vaccine dose. Grey shading indicates projected vaccine effectiveness beyond the time period of the data to which the model was fitted.

Vaccine	Days post dose 2		Days post dose 3						
	90	180	30	60	90	120	150	180	365
Mild disease									
Oxford/AstraZeneca AZD1222	56.5 (55.7-57.2)	38.3 (37-39.2)	87.5 (84.1-90.1)	80.2 (75.4-84.1)	72.4 (66.4-77.3)	65.3 (58.7-71)	59.7 (52.5-65.8)	55.6 (48.2-61.9)	43.7 (35.3-51)
Moderna mRNA-1273	82.7 (81.9-83.1)	69.5 (67.9-70.4)	94.4 (94.1-94.6)	90.7 (90.2-91)	86.2 (85.5-86.7)	81.8 (80.8-82.4)	78 (76.7-78.8)	74.9 (73.4-75.9)	65.1 (60-67.7)
Pfizer-BioNTech BNT162b2	76 (75.3-76.3)	60.1 (58.8-60.8)	92.7 (92.4-92.9)	88 (87.5-88.3)	82.6 (81.9-83)	77.3 (76.4-77.9)	72.8 (71.7-73.6)	69.4 (67.8-70.3)	58.5 (53.1-61.3)
Hospitalisation									
Oxford/AstraZeneca AZD1222	90.7 (90.2-91.1)	82.4 (81.1-83.2)	98.1 (97.6-98.6)	96.8 (95.9-97.6)	95.2 (93.7-96.3)	93.4 (91.4-94.9)	91.8 (89.2-93.6)	90.4 (87.4-92.5)	85.4 (80-88.8)
Moderna mRNA-1273	97.3 (97.1-97.5)	94.5 (93.9-94.8)	99.2 (99.1-99.3)	98.7 (98.5-98.7)	97.9 (97.7-98)	97.1 (96.9-97.3)	96.4 (96-96.6)	95.7 (95.3-96.1)	93.3 (91.7-94.2)
Pfizer-BioNTech BNT162b2	96 (95.7-96.2)	91.9 (91.2-92.3)	99 (98.9-99)	98.2 (98.1-98.3)	97.3 (97.1-97.4)	96.2 (95.9-96.4)	95.3 (94.9-95.5)	94.5 (93.9-94.8)	91.4 (89.3-92.4)
Death									
Oxford/AstraZeneca AZD1222	90.5 (89.1-91.7)	82 (79.5-84.1)	98.1 (97.3-98.6)	96.8 (95.5-97.6)	95.1 (93.2-96.3)	93.2 (90.7-94.9)	91.6 (88.5-93.6)	90.2 (86.5-92.5)	85.1 (79.5-88.6)
Moderna mRNA-1273	97.2 (96.7-97.6)	94.3 (93.3-95.1)	99.2 (99-99.3)	98.6 (98.4-98.8)	97.9 (97.5-98.1)	97.1 (96.5-97.4)	96.3 (95.6-96.8)	95.6 (94.8-96.2)	93.2 (91.3-94.3)
Pfizer-BioNTech BNT162b2	95.9 (95.2-96.4)	91.7 (90.3-92.8)	98.9 (98.7-99.1)	98.2 (97.8-98.4)	97.2 (96.7-97.6)	96.2 (95.4-96.6)	95.2 (94.3-95.8)	94.3 (93.3-95)	91.2 (88.8-92.7)

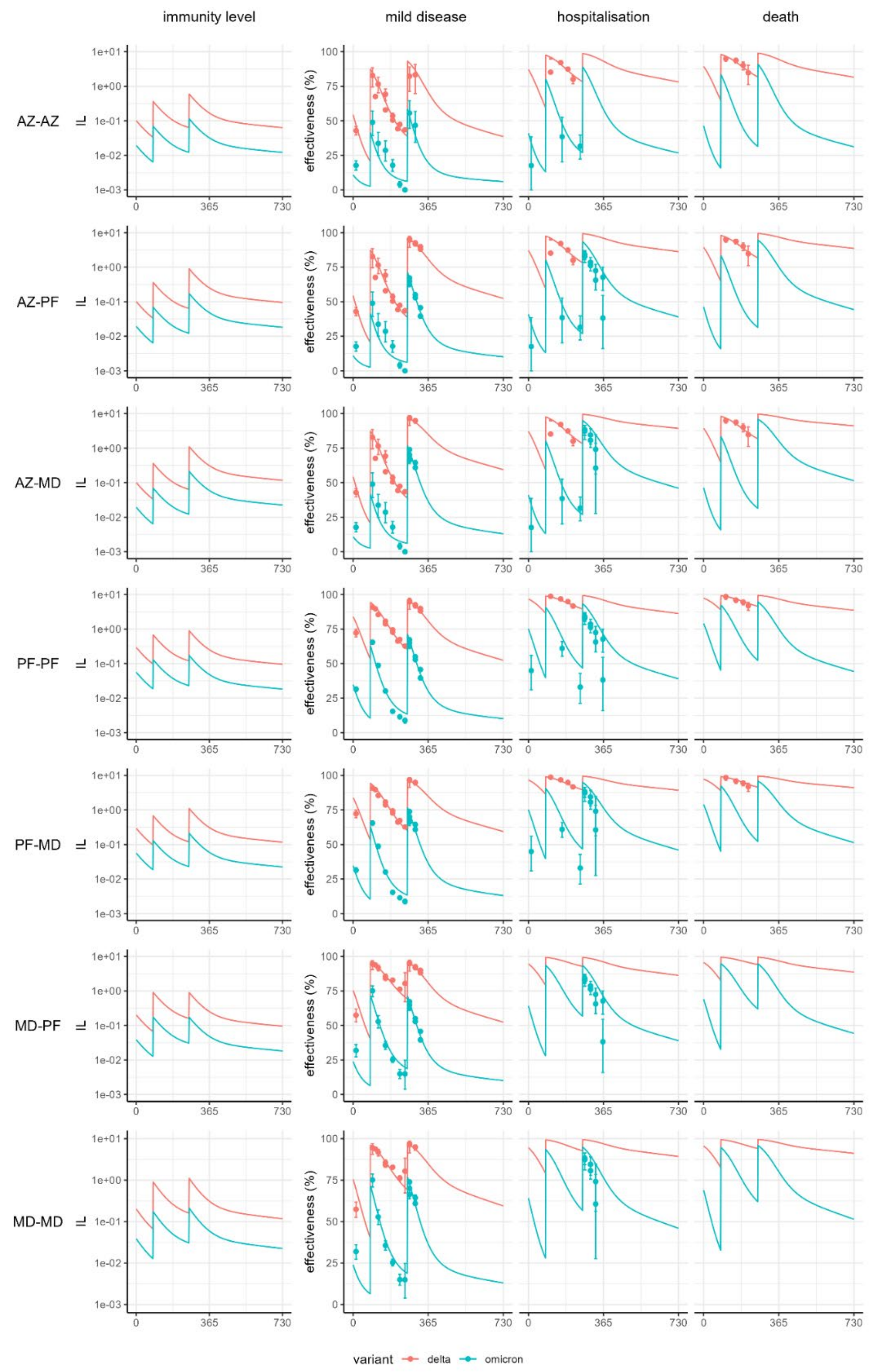

Figure S1: Projected vaccine effectiveness over time (in days) since the first vaccine dose for combinations of schedules for the Oxford/AstraZeneca AZD1222 (AZ), Pfizer-BioNTech BNT162b2 (PF) and Moderna mRNA-1273 (MD) vaccines using data from 18-64 age-group where only stratified data were available. Plots show immunity level (IL) in the left column, alongside effectiveness against mild disease, hospitalisation, and death on the right. Neutralization and protection against the Delta and Omicron variants are shown in red and blue respectively. Seven regimens are shown: AZ delivered for three doses (AZ-AZ); two doses of AZ and a third dose of PF (AZ-PF); two doses of AZ and a third dose of MD (AZ-MD); PF delivered for three doses (PF-PF); two doses of PF and a third dose of MD (PF-MD); two doses of MD and a third dose of PF (MD-PF); and MD delivered for three doses (MD-MD). The solid lines show the posterior median fitted model estimate, and the points show estimates of mean vaccine effectiveness (and associated 95% confidence intervals) against three endpoints using data from England[24–26]. Sample sizes for each of the 143 data points vary according to the uptake of combinations and length of follow-up; these are provided in the data files.

Table S2: Estimated vaccine effectiveness against mild disease, hospitalisation and death for Oxford/AstraZeneca AZD1222, Pfizer-BioNTech BNT162b2 and Moderna mRNA-1273 vaccine regimens as a function of time since dose 2 or dose 3. Estimates are shown for the Omicron variant using the data from the 18-65 age-group where only age-stratified data were available. Values shown are the posterior median and 95% credible intervals. The comparator group is those that did not receive any vaccine dose.

Vaccine	Days post dose 2		Days post dose 3						
	90	180	30	60	90	120	150	180	365
Mild disease									
Oxford/AstraZeneca AZD1222	12.1 (11.7-12.6)	6 (5.7-6.3)	42.1 (35.7-48.8)	29.8 (24.6-35.6)	21.5 (17.3-26.2)	16.3 (12.9-20.3)	13.2 (10.4-16.5)	11.3 (8.8-14.2)	7.1 (5.1-9.3)
Moderna mRNA-1273	32.9 (31.9-33.9)	18.6 (17.5-19.4)	63.1 (62.2-63.8)	49.9 (48.7-50.9)	39.1 (37.8-40.2)	31.4 (30.2-32.5)	26.3 (25.1-27.3)	23 (21.6-24)	15.2 (12.5-17.1)
Pfizer-BioNTech BNT162b2	24.8 (24.1-25.3)	13.3 (12.6-13.8)	56.2 (55.6-56.7)	42.8 (42.1-43.5)	32.5 (31.8-33.3)	25.6 (24.8-26.4)	21.1 (20.3-21.9)	18.3 (17.4-19.1)	11.9 (9.7-13.4)
Hospitalisation									
Oxford/AstraZeneca AZD1222	44 (42.7-45.5)	26.7 (25.3-28.1)	80.5 (76-84.6)	70.7 (64.8-76.1)	60.8 (54.1-67.2)	52.5 (45.7-59.3)	46.3 (39.5-53)	41.9 (35.2-48.5)	30.2 (23.2-37)
Moderna mRNA-1273	73.6 (72.4-74.8)	56.4 (54.4-58.3)	90.7 (90.2-91.1)	85 (84.2-85.7)	78.5 (77.4-79.5)	72.2 (70.8-73.6)	67 (65.3-68.6)	62.9 (60.9-64.7)	50.5 (44.7-54.3)
Pfizer-BioNTech BNT162b2	65.2 (64-66.4)	46.5 (44.7-48.2)	87.9 (87.4-88.5)	81 (80.2-81.7)	73.3 (72.3-74.3)	66.1 (64.9-67.4)	60.4 (58.8-61.9)	56 (54.1-57.8)	43.3 (37.8-47.2)
Death									
Oxford/AstraZeneca AZD1222	49.3 (45.7-53.5)	31.1 (27.9-34.7)	83.7 (79.1-87.5)	74.9 (68.8-80.3)	65.8 (58.6-72.6)	57.9 (50.2-65.5)	51.7 (43.9-59.6)	47.2 (39.4-55.2)	35 (26.7-43.5)
Moderna mRNA-1273	77.6 (75-80.4)	61.6 (57.9-65.5)	92.3 (91.2-93.4)	87.5 (85.8-89.2)	81.9 (79.5-84.2)	76.4 (73.5-79.1)	71.6 (68.3-74.7)	67.8 (64.1-71.1)	55.8 (49.4-61.1)
Pfizer-BioNTech BNT162b2	69.9 (66.7-73.2)	51.9 (48-55.9)	90 (88.7-91.4)	84.1 (82.1-86.2)	77.3 (74.6-80.1)	70.8 (67.6-74.2)	65.4 (61.8-69.1)	61.2 (57.4-65.2)	48.7 (42.4-54.2)

Table S3: Statistical fit of the main model and the two sensitivity analyses to assumed model structure. Statistics are reported from the sampling phase of the MCMC chain discarding the burn-in period.

Model	Number of parameters	LogLikelihood (Mean)	LogLikelihood (SE)
Main	17	-79,474	3.05
Alternative Severity	17	-79,464	3.03
Additive Boost	17	-79,849	3.30

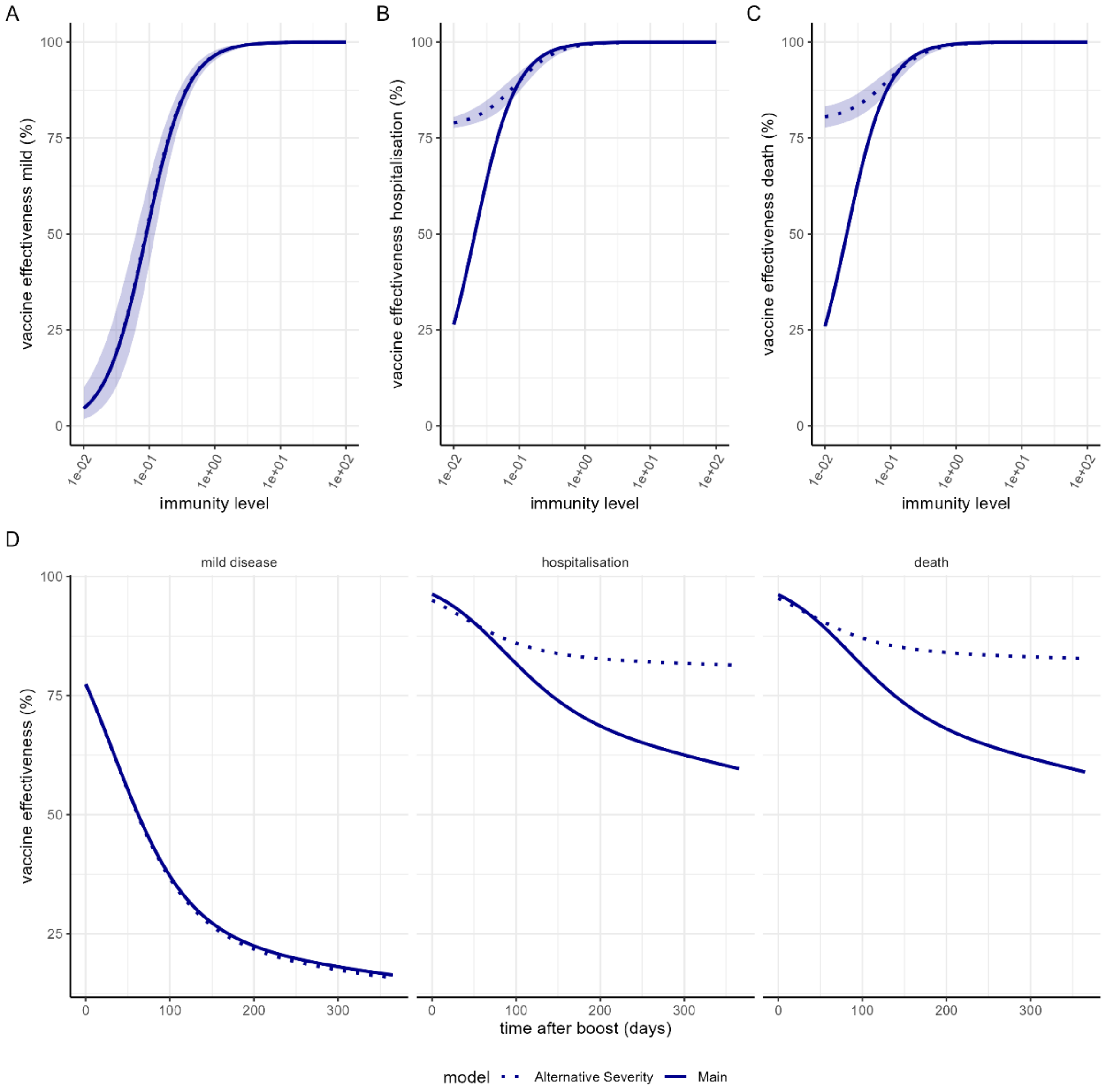

Figure S2: Fitted relationships for the alternative severity model. Dose-response curves estimated from fitting to vaccine effectiveness data for the relationship between immunity level (IL, x-axis) and vaccine effectiveness against mild disease (A), hospitalisation (B) and death (C) against the Omicron/BA.1 variant. The dashed lines show the posterior median estimates and colour bands the 95% credible interval. The solid lines show the dose-response curves using the main model. (D) Projected vaccine effectiveness for the Moderna mRNA-1273 vaccine from time since dose 3 for the main model (solid line) and the alternative severity model (dashed line). The values are for the posterior median estimates.

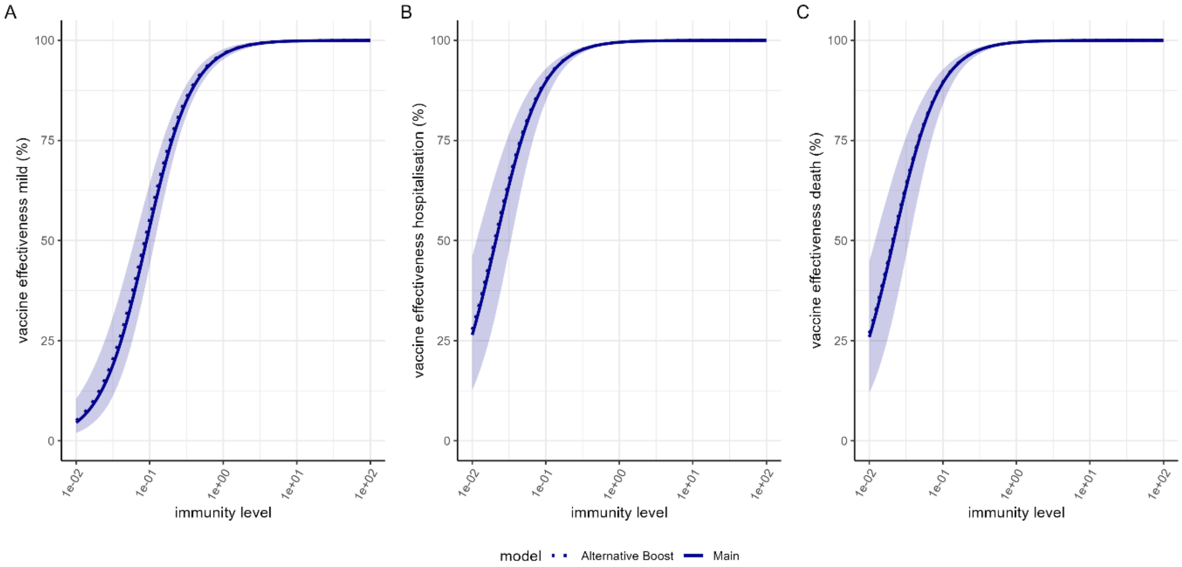

Figure S3: Fitted relationships for the additive boosting model. Dose-response curves estimated from fitting to vaccine effectiveness data for the relationship between immunity level (IL, x-axis) and vaccine effectiveness against mild disease (A), hospitalisation (B) and death (C) against the Omicron/BA.1 variant. The dashed lines show the posterior median estimates and colour bands the 95% credible interval. The solid lines show the dose-response curves using the main model. Given the overlap between the estimated relationships, vaccine effectiveness estimates are similar to those from the main model.

Table S4: **Estimated vaccine effectiveness against mild disease, hospitalisation and death for Oxford/AstraZeneca AZD1222, Pfizer-BioNTech BNT162b2 and Moderna mRNA-1273 vaccine regimens as a function of time since dose 2 or dose 3.** Estimates are shown for the Omicron variant with the ancestral vaccines for the alternative severity model. The values are shown for each vaccine for dose 2 or dose 3; as the dose 3 estimates do not depend on dose 2 values, the estimates are applicable to either homologous or heterologous dosing. Values shown are the posterior median and 95% credible intervals. The comparator group is those who did not receive any vaccine dose. Grey shading indicates projected vaccine effectiveness beyond the time period of the data to which the model was fitted.

**Vaccine**	**Days post dose 2**		**Days post dose 3**						
	**90**	**180**	**30**	**60**	**90**	**120**	**150**	**180**	**365**
Mild disease									
Oxford/AstraZeneca AZD1222	11.9 (11.4-12.3)	5.9 (5.6-6.2)	42.1 (35.4-49.1)	29.5 (24-35.9)	21.2 (16.7-26.5)	16.1 (12.5-20.4)	13 (10.1-16.7)	11.2 (8.6-14.5)	7.2 (5.1-9.5)
Moderna mRNA-1273	33 (31.8-33.9)	18.7 (17.5-19.6)	63.6 (62.5-64.2)	50.2 (48.8-51)	39.2 (37.8-40.2)	31.5 (30.2-32.5)	26.5 (25.1-27.4)	23.2 (21.8-24.3)	15.7 (12.7-17.5)
Pfizer-BioNTech BNT162b2	24.8 (24-25.3)	13.4 (12.6-13.8)	56.6 (55.8-57)	42.9 (41.9-43.5)	32.5 (31.4-33.2)	25.6 (24.5-26.2)	21.2 (20.1-21.9)	18.4 (17.2-19.2)	12.2 (9.8-13.8)
Hospitalisation									
Oxford/AstraZeneca AZD1222	80.5 (79.5-81.6)	79.2 (78.1-80.3)	87.2 (85.6-88.8)	84.4 (83-86)	82.6 (81.2-84)	81.5 (80.2-82.7)	80.8 (79.6-82)	80.4 (79.2-81.6)	79.5 (78.3-80.6)
Moderna mRNA-1273	85.2 (84.3-86)	82.1 (81-83)	92 (91.4-92.4)	89 (88.3-89.6)	86.6 (85.8-87.3)	84.9 (84-85.7)	83.8 (82.8-84.6)	83 (82-84)	81.4 (80.1-82.4)
Pfizer-BioNTech BNT162b2	83.4 (82.4-84.2)	80.9 (79.8-81.9)	90.4 (89.8-90.9)	87.4 (86.7-88)	85.1 (84.2-85.8)	83.6 (82.6-84.4)	82.6 (81.6-83.5)	82 (80.9-82.9)	80.6 (79.4-81.7)
Death									
Oxford/AstraZeneca AZD1222	82 (79.4-84.2)	80.8 (78-83.2)	88.2 (85.8-90.2)	85.6 (83.1-87.8)	83.9 (81.3-86.1)	82.9 (80.2-85.1)	82.3 (79.4-84.6)	81.9 (79-84.2)	81.1 (78.1-83.5)
Moderna mRNA-1273	86.3 (84.3-88)	83.4 (80.9-85.4)	92.6 (91.4-93.5)	89.8 (88.3-91.1)	87.6 (85.7-89.1)	86 (83.9-87.7)	85 (82.7-86.9)	84.3 (81.9-86.3)	82.8 (80-84.9)
Pfizer-BioNTech BNT162b2	84.7 (82.3-86.5)	82.3 (79.7-84.5)	91.2 (89.8-92.2)	88.4 (86.6-89.7)	86.2 (84.2-87.9)	84.8 (82.5-86.6)	83.9 (81.5-85.9)	83.4 (80.8-85.4)	82.1 (79.3-84.3)

Table S5: **Estimated vaccine effectiveness against mild disease, hospitalisation and death for Oxford/AstraZeneca AZD1222, Pfizer-BioNTech BNT162b2 and Moderna mRNA-1273 vaccine regimens as a function of time since dose 2 or dose 3.** Estimates are shown for the Omicron variant with the ancestral vaccines for the additive boosting model. The values are shown for each vaccine for dose 2 or dose 3; as the dose 3 estimates do not depend on dose 2 values, the estimates are applicable to either homologous or heterologous dosing. Values shown are the posterior median and 95% credible intervals. The comparator group is those who did not receive any vaccine dose. Grey shading indicates projected vaccine effectiveness beyond the time period of the data to which the model was fitted.

**Vaccine**	**Days post dose 2**		**Days post dose 3**						
	**90**	**180**	**30**	**60**	**90**	**120**	**150**	**180**	**365**
Mild disease									
Oxford/AstraZeneca AZD1222	15.1 (14.5-15.5)	7.5 (7-7.8)	71.4 (61-80.4)	58.8 (47.3-70.2)	47.5 (36.3-60)	39 (28.6-51.4)	33.1 (23.8-44.9)	29.2 (20.6-40.4)	20.2 (13.1-29)
Moderna mRNA-1273	34.1 (33-35)	19 (17.9-19.9)	82.1 (75.8-87.4)	72.4 (64.1-80)	62.5 (53.2-71.8)	54 (44.2-64.2)	47.6 (38-58.1)	43.1 (33.9-53.4)	31.7 (24-40.6)
Pfizer-BioNTech BNT162b2	23.3 (22.6-23.8)	12.1 (11.4-12.6)	77.7 (70.4-84)	66.6 (57.5-75.1)	55.8 (46.1-65.9)	47.1 (37.6-57.7)	40.8 (31.8-51.2)	36.5 (28.1-46.2)	26.1 (19.2-33.8)
Hospitalisation									
Oxford/AstraZeneca AZD1222	56.3 (54.5-57.7)	36.9 (34.8-38.5)	94.8 (91.9-96.8)	91.2 (86.7-94.5)	86.8 (80.4-91.6)	82.2 (74.1-88.5)	78.2 (69-85.7)	74.9 (65-83.1)	64.7 (52.4-74.5)
Moderna mRNA-1273	78.9 (77.5-80)	63 (60.6-65)	97.1 (95.8-98.1)	95 (92.8-96.7)	92.4 (89.1-94.9)	89.5 (85.1-92.9)	86.8 (81.6-90.9)	84.6 (78.8-89.2)	77.1 (69.2-83.2)
Pfizer-BioNTech BNT162b2	68.7 (67.2-69.9)	50 (47.6-51.6)	96.2 (94.5-97.4)	93.5 (90.7-95.7)	90.2 (86-93.3)	86.6 (81.3-90.8)	83.3 (77.1-88.3)	80.6 (73.6-86.1)	71.9 (63.1-78.8)
Death									
Oxford/AstraZeneca AZD1222	55.1 (51.5-58.7)	35.8 (32.1-39.5)	94.5 (91.5-96.8)	90.8 (86-94.5)	86.2 (79.5-91.5)	81.5 (73.1-88.3)	77.4 (67.8-85.4)	74 (63.6-83)	63.6 (51.4-74.7)
Moderna mRNA-1273	78.2 (75.4-80.6)	61.9 (57.7-65.6)	96.9 (95.5-98)	94.8 (92.4-96.6)	92 (88.5-94.7)	89 (84.4-92.7)	86.3 (80.7-90.6)	84 (77.7-88.9)	76.3 (68.1-82.8)
Pfizer-BioNTech BNT162b2	67.7 (64.3-70.8)	48.8 (44.6-52.7)	96 (94.1-97.4)	93.2 (90.2-95.5)	89.7 (85.3-93.1)	86 (80.3-90.5)	82.7 (75.8-88)	79.9 (72.5-85.7)	70.9 (61.6-78.4)

Table S6: **Projected vaccine effectiveness against mild disease, hospitalisation and death from BA.1 for an ancestral vaccine (BNT162b2) and variant-adapted vaccine as a function of time since a fourth dose.** Values shown are the posterior median and 95% credible intervals. The comparator group is those that did not receive any vaccine dose.

Vaccine	Days post 4th dose						
	30	60	90	120	150	180	365
Mild disease							
Pfizer-BioNTech BNT162b2	57.1 (56.2-57.4)	43.5 (42.5-44.1)	33.2 (32.1-33.9)	26.3 (25.3-27)	22 (20.9-22.6)	19.2 (18.1-19.9)	12.9 (10.6-14.2)
Variant-adapted vaccine	74.3 (68-76.4)	62.6 (55.1-65.2)	51.9 (44.1-54.8)	43.7 (36.1-46.7)	37.9 (30.7-40.8)	34 (27.2-36.7)	24.3 (17.9-27.1)
Hospitalisation							
Pfizer-BioNTech BNT162b2	90.9 (90.3-91.3)	85.3 (84.3-85.9)	78.9 (77.6-79.8)	72.9 (71.2-74)	67.9 (65.8-69.2)	64.1 (61.7-65.7)	52.6 (46.9-56)
Variant-adapted vaccine	95.6 (94.1-96.1)	92.6 (90.2-93.4)	89 (85.5-90.2)	85.4 (80.9-86.9)	82.1 (76.9-83.8)	79.5 (73.7-81.4)	70.7 (61.8-73.7)
Death							
Pfizer-BioNTech BNT162b2	90.7 (89.1-91.8)	85 (82.7-86.6)	78.5 (75.5-80.8)	72.4 (68.8-75.2)	67.4 (63.4-70.5)	63.5 (59.3-66.9)	52 (45.4-56.6)
Variant-adapted vaccine	95.5 (93.6-96.1)	92.5 (89.6-93.5)	88.8 (84.7-90.4)	85 (79.9-87.1)	81.7 (75.7-84.2)	79.1 (72.5-81.8)	70.2 (60.6-73.9)

Table S7: **Projected vaccine effectiveness against mild disease, hospitalisation and death from BA.1 for an ancestral vaccine (AZD1222) and variant-adapted vaccine as a function of time since a fourth dose.** Values shown are the posterior median and 95% credible intervals. The comparator group is those that did not receive any vaccine dose.

Vaccine	Days post 4th dose						
	30	60	90	120	150	180	365
Mild disease							
Oxford/AstraZeneca AZD1222	42.4 (35.8-48.9)	29.9 (24.4-35.7)	21.6 (17.2-26.4)	16.5 (13-20.4)	13.4 (10.4-16.8)	11.6 (8.9-14.5)	7.5 (5.4-9.8)
Variant-adapted vaccine	61.5 (51.6-67.3)	48 (38.2-54.4)	37.4 (28.5-43.5)	30 (22.3-35.5)	25.2 (18.3-30.1)	22.2 (15.8-26.6)	15 (9.8-18.8)
Hospitalisation							
Oxford/AstraZeneca AZD1222	84.7 (80.7-88)	76.2 (70.8-80.8)	67.4 (61-73.1)	59.8 (52.6-66.2)	53.9 (46.6-60.6)	49.7 (42.2-56.5)	38 (29.8-45.3)
Variant-adapted vaccine	92.3 (88.8-94)	87.4 (82.2-90.1)	81.8 (75-85.4)	76.3 (68.2-80.6)	71.7 (62.8-76.5)	68.2 (58.5-73.3)	57.1 (44.8-63.4)
Death							
Oxford/AstraZeneca AZD1222	84.4 (79.3-87.8)	75.8 (68.9-80.7)	66.8 (58.9-72.9)	59.1 (50.8-66)	53.3 (44.6-60.5)	49 (40.3-56.3)	37.4 (28.8-44.8)
Variant-adapted vaccine	92.1 (88.2-94)	87.1 (81.2-90.2)	81.4 (73.4-85.6)	75.9 (66.5-81)	71.2 (61-77.1)	67.6 (56.8-73.9)	56.5 (43.7-64)
