# Supplementary material for: Estimating long-term vaccine effectiveness against SARS-CoV-2 variants: a model-based approach
Source: Nat Commun. 2023 Jul 19;14:4325. doi: 10.1038/s41467-023-39736-3 (PMC10356855; doi:10.1038/s41467-023-39736-3)
Supplement: Supplementary file 3 — Reporting Summary [file 41467_2023_39736_MOESM3_ESM.pdf]

nature portfolio

Corresponding author(s): Azra Ghani

Last updated by author(s): 2023/06/14

Reporting Summary

Nature Portfolio wishes to improve the reproducibility of the work that we publish. This form provides structure for consistency and transparency in reporting. For further information on Nature Portfolio policies, see our Editorial Policies and the Editorial Policy Checklist.

Statistics

For all statistical analyses, confirm that the following items are present in the figure legend, table legend, main text, or Methods section.

n/a	Confirmed	
☒	☐	The exact sample size (n) for each experimental group/condition, given as a discrete number and unit of measurement
☒	☐	A statement on whether measurements were taken from distinct samples or whether the same sample was measured repeatedly
☒	☐	The statistical test(s) used AND whether they are one- or two-sided<br>*Only common tests should be described solely by name; describe more complex techniques in the Methods section.*
☒	☐	A description of all covariates tested
☐	☒	A description of any assumptions or corrections, such as tests of normality and adjustment for multiple comparisons
☐	☒	A full description of the statistical parameters including central tendency (e.g. means) or other basic estimates (e.g. regression coefficient) AND variation (e.g. standard deviation) or associated estimates of uncertainty (e.g. confidence intervals)
☒	☐	For null hypothesis testing, the test statistic (e.g. F, t, r) with confidence intervals, effect sizes, degrees of freedom and P value noted<br>*Give P values as exact values whenever suitable.*
☐	☒	For Bayesian analysis, information on the choice of priors and Markov chain Monte Carlo settings
☒	☐	For hierarchical and complex designs, identification of the appropriate level for tests and full reporting of outcomes
☒	☐	Estimates of effect sizes (e.g. Cohen's d, Pearson's r), indicating how they were calculated

Our web collection on statistics for biologists contains articles on many of the points above.

Software and code

Policy information about availability of computer code

Data collection	Summary data (vaccine efficacy estimates) were obtained from published papers and entered into a text file using Microsoft® Excel® for Microsoft 365 MSO (Version 2304 Build 16.0.16327.20200) 64-bit. The summary data used in the fitting are provided in the Github repo.
Data analysis	Custom open source R code (run using version 4.3.0) was used in the fitting utilising freely available R packages. All code is provided in Github at https://github.com/mrc-ide/covid_efficacy. Some outputs from the code could not be uploaded due to file size limits - these can be generated from the provided code but can also be shared on request.

For manuscripts utilizing custom algorithms or software that are central to the research but not yet described in published literature, software must be made available to editors and reviewers. We strongly encourage code deposition in a community repository (e.g. GitHub). See the Nature Portfolio guidelines for submitting code & software for further information.

Data

Policy information about availability of data

All manuscripts must include a data availability statement. This statement should provide the following information, where applicable:

- Accession codes, unique identifiers, or web links for publicly available datasets
- A description of any restrictions on data availability
- For clinical datasets or third party data, please ensure that the statement adheres to our policy

The data used in the fitting were extracted from the cited papers and are provided at: https://github.com/mrc-ide/covid_efficacy

Human research participants

Policy information about studies involving human research participants and Sex and Gender in Research.

Reporting on sex and gender	Data were not reported by sex or gender in the original publications and so this could not be included in this analysis.
Population characteristics	Provided in the original reporting manuscript
Recruitment	N/A - population based data from national surveillance
Ethics oversight	Not required - only utilises summary estimates available in the public domain.

Note that full information on the approval of the study protocol must also be provided in the manuscript.

Field-specific reporting

Please select the one below that is the best fit for your research. If you are not sure, read the appropriate sections before making your selection.

☒ Life sciences ☐ Behavioural & social sciences ☐ Ecological, evolutionary & environmental sciences

For a reference copy of the document with all sections, see nature.com/documents/nr-reporting-summary-flat.pdf

Life sciences study design

All studies must disclose on these points even when the disclosure is negative.

Sample size	The study relies on previously published summary estimates and associated confidence interval. The data in this study were obtained from observational follow-up of a national cohort for England, representing the population of ~50 million people. The resulting precision of estimates of vaccine efficacy depend on both the denominator population (which is large) but also the number of COVID-19 events. In some age-groups this is small, and this lack of precision is reflected in the associated confidence intervals. This uncertainty is propagated through to the model-estimated parameters and their uncertainty intervals.
Data exclusions	None
Replication	Replication is not applicable to this study; the data are from a national cohort which is not an experiment.
Randomization	The study data used in the secondary analysis was not randomised as it is an observational cohort following outcomes in those who did and didn't receive the COVID-19 vaccines. It would not have been possible or ethical to randomise to receiving a vaccine in the context of an ongoing pandemic with associated health risks.
Blinding	Data are from an observational cohort derived from national public health records. As this was not a randomized study, participants were not blinded to the vaccine that they received and no placebo doses were administered.

Reporting for specific materials, systems and methods

We require information from authors about some types of materials, experimental systems and methods used in many studies. Here, indicate whether each material, system or method listed is relevant to your study. If you are not sure if a list item applies to your research, read the appropriate section before selecting a response.

Materials & experimental systems

n/a	Involved in the study	
☒	☐	Antibodies
☒	☐	Eukaryotic cell lines
☒	☐	Palaeontology and archaeology
☒	☐	Animals and other organisms
☒	☐	Clinical data
☐	☒	Dual use research of concern

Methods

n/a	Involved in the study	
☒	☐	ChIP-seq
☒	☐	Flow cytometry
☒	☐	MRI-based neuroimaging

Dual use research of concern

Policy information about dual use research of concern

Hazards

Could the accidental, deliberate or reckless misuse of agents or technologies generated in the work, or the application of information presented in the manuscript, pose a threat to:

No	Yes	
☒	☐	Public health
☒	☐	National security
☒	☐	Crops and/or livestock
☒	☐	Ecosystems
☒	☐	Any other significant area

Experiments of concern

Does the work involve any of these experiments of concern:

No	Yes	
☒	☐	Demonstrate how to render a vaccine ineffective
☒	☐	Confer resistance to therapeutically useful antibiotics or antiviral agents
☒	☐	Enhance the virulence of a pathogen or render a nonpathogen virulent
☒	☐	Increase transmissibility of a pathogen
☒	☐	Alter the host range of a pathogen
☒	☐	Enable evasion of diagnostic/detection modalities
☒	☐	Enable the weaponization of a biological agent or toxin
☒	☐	Any other potentially harmful combination of experiments and agents
